# Supplementary material for: Resolving optimal ionomer interaction in fuel cell electrodes via operando X-ray absorption spectroscopy
Source: Nat Commun. 2024 Oct 30;15:9390. doi: 10.1038/s41467-024-53823-z (PMC11525635; doi:10.1038/s41467-024-53823-z)
Supplement: Supplementary file 1 — Supplementary Information [file 41467_2024_53823_MOESM1_ESM.pdf]

Supplementary Information for

Resolving Optimal Ionomer Interaction in Fuel Cell Electrodes via operando X-ray Absorption Spectroscopy

Mengnan Wang[1,2], Jiaguang Zhang[3], Silvia Favero[1], Luke J.R. Higgins[4], Hui Luo[1], Ifan.E.L. Stephens *[2], Maria Magdalena Titirici[1,5]*

[1]Department of Chemical Engineering, Imperial College London, South Kensington Campus SW7 2AZ London, UK

[2]Department of Materials, Imperial College London. White City Campus, 80 Wood Ln, London, W12 7TA, UK

[3]Department of Chemistry, University of Lincoln, Bayford Pool, Lincoln LN6 7TS, UK

[4]Diamond Light Source Ltd, Diamond House, Harwell Science & Innovation Campus, Didcot Oxfordshire, OX11 0DE, UK

[5]Advanced Institute for Materials Research (AIMR), Tohoku University, 2-1-1 Katahira, Aoba-ku, Sendai, 980-8577 Japan

* i.stephens@imperial.ac.uk

* m.titirici@imperial.ac.uk

Calculation for fully utilised the mesoporous channels

Loading of the Pt is targeted at 1 wt% based on below estimation to fully utilised the mesoporous channels in HOMC.

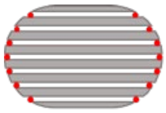

$$Pt\ wt\% = \frac{mass\ of\ Pt\ NPs\ on\ one\ HOMC}{mass\ of\ carbon\ in\ one\ HOMC} = \frac{No.\ of\ channels \times 2 \times V_{PtNP} \times d_{Pt}}{Volume\ of\ channels \div specific\ mesopore\ volume}$$

$$\approx 0.2\ wt\% \sim 1\ wt\%$$

$No.\ of\ channels$ calculated from the [distance between the ordered mesopores] and [size of the MCS] from TEM

V_{PtNP} Volume of each Pt NP, calculated from the average size of Pt NP from TEM

$Volume\ of\ channels$ calculated from the [average length] and [diameter] of the ordered mesopores and $No.\ of\ channels$ from TEM

$specific\ mesopore\ volume$ obtained from accumulative specific pore volume plot in the range of 2-4 nm from BET

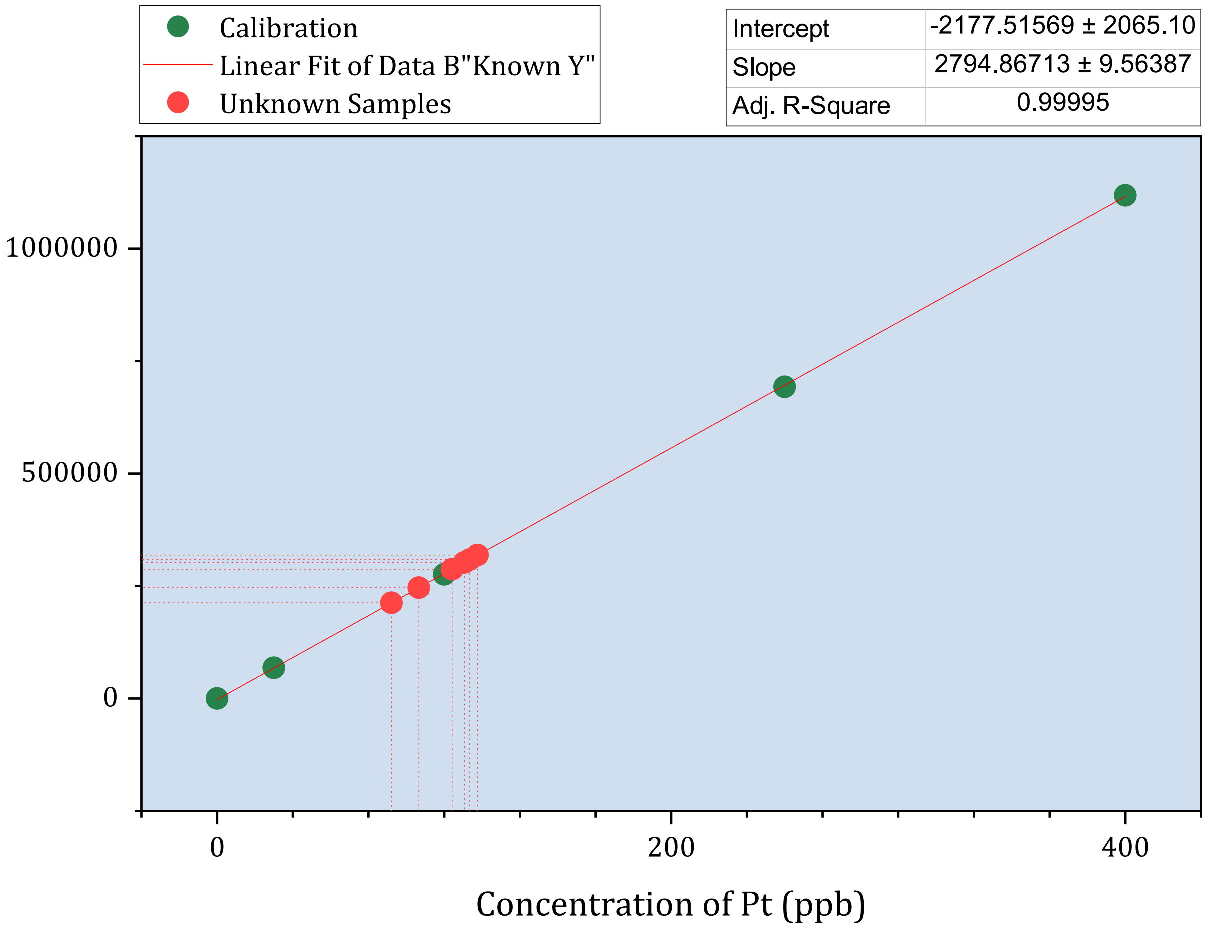

	Pt concentration (ppb)	counts per second	
Calibration results	0	81.75667	
	25	68185.56667	
	100	275613.1633	
	250	692678.1	
	400	1118575.863	
	**Pt concentration after 35 times dilution (ppb)**	**counts per second**	**Weight percentage of Pt**
Pt/HOMC	103.48679	287054.3133	0.72%
	111.21035	308640.6293	0.78%
Pt/Vulcan	114.71033	318422.6077	0.80%
	108.86451	302084.3133	0.76%
Pt/Ketjenblack	88.83651	246108.7267	0.62%
	76.77626	212401.9333	0.54%

Figure S1. ICP results with calibration results. Showing the weight percentage of Pt for all 3 catalysts.

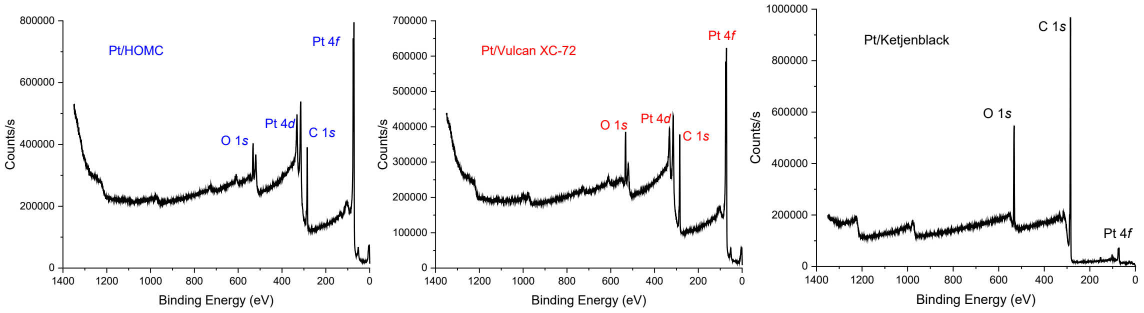

Figure S2. XPS survey of the 3 Pt/C catalysts.

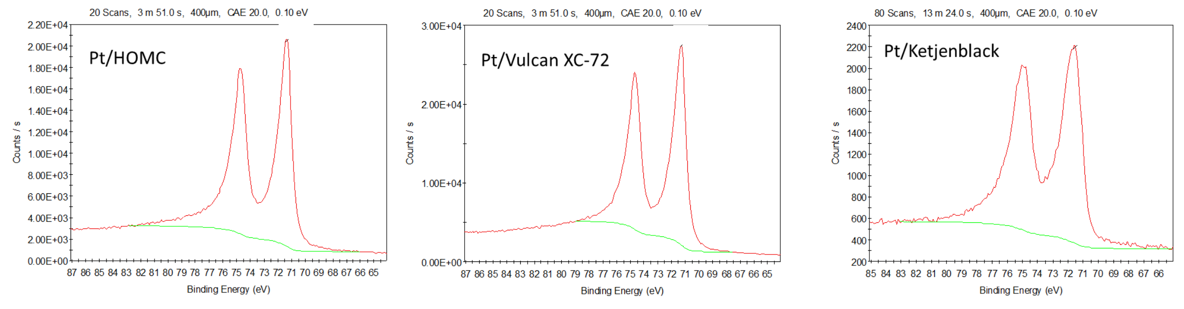

Figure S3. XPS Spectra of Pt/HOMC, Pt/Vulcan XC-72 and Pt/Ketjenblack indicating similar electronic properties of obtained Pt NPs.

Table S1. Bulk Pt content measured via ICP and surface Pt content measured via XPS for the 3 catalysts. The weight ratios were derived from XPS atom ratios using standard atomic weights.

	**Pt/Ketjenblack**	**Pt/Vulcan**	**Pt/HOMC**
Pt content by ICP	0.58 wt%	0.78 wt%	0.75 wt%
Surface Pt content by XPS	0.49 wt%	14.98 wt%	12.6 wt%

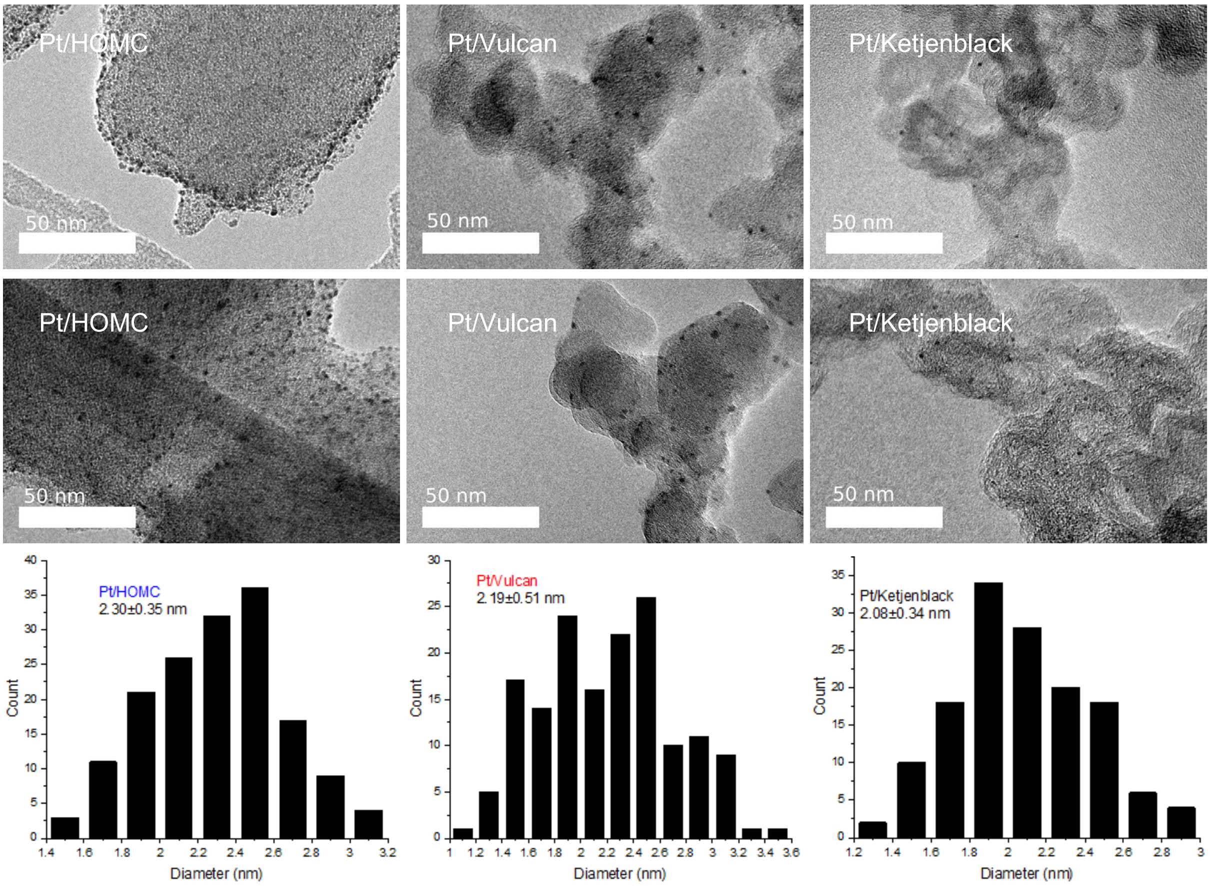

Figure S4. TEM images of Pt/HOMC, Pt/Vulcan XC-72 and Pt/Ketjenblack indicating similar particles sized obtained for 3 catalysts.

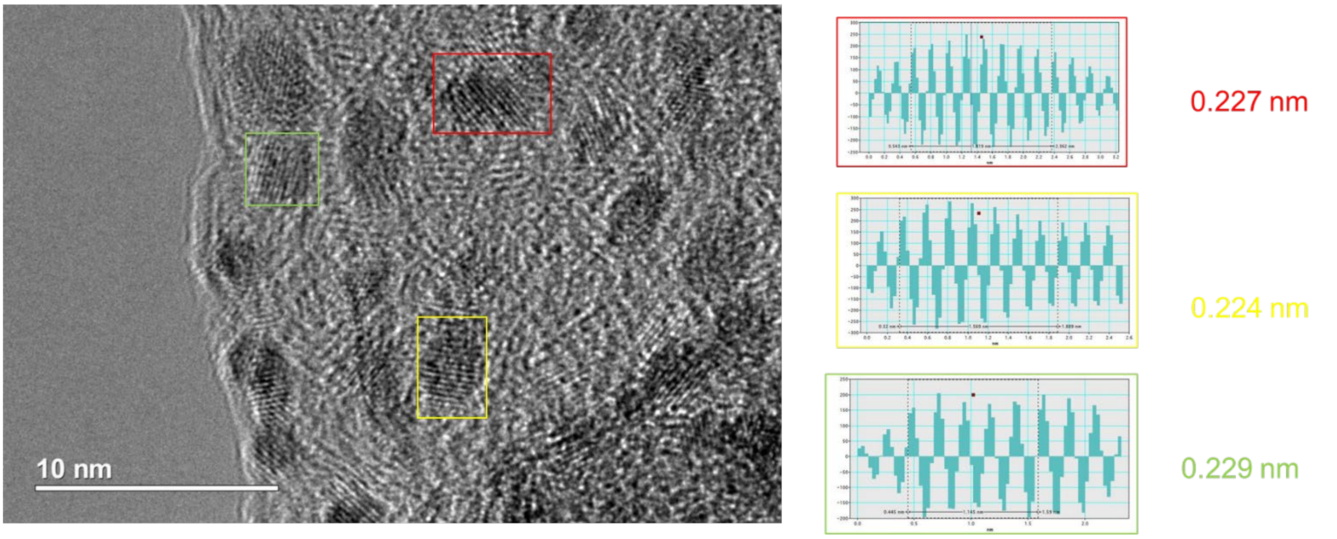

Figure S5. HRTEM image of Pt/HOMC reveals lattice fringe spacings corresponding to the Pt (111) plane.

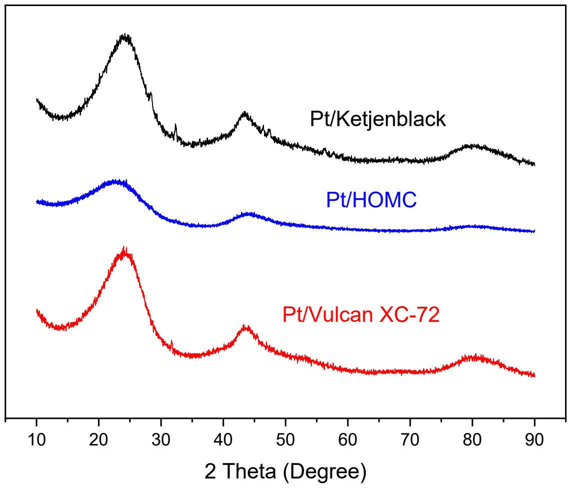

Figure S6. XRD patterns of the three catalysts with no visible Pt peaks due to the low Pt loading.

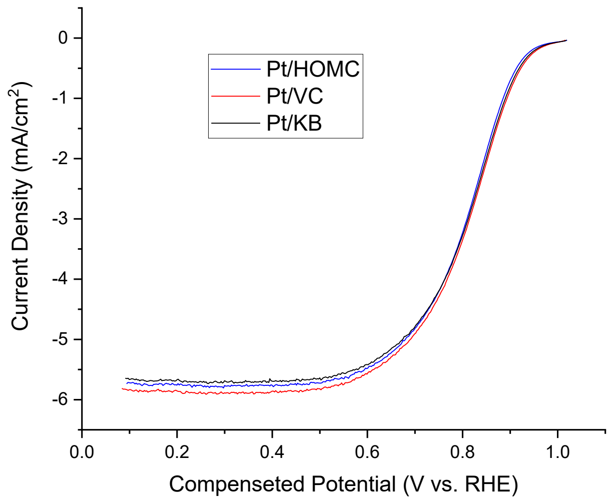

Figure S7. ORR polarization curves measured in RDE half-cell. Measurements were done in 1600 rpm, 0.1M $HClO_4$ electrolyte at room temperature with 200 mL/min oxygen flow and 20 mV/s cathodic scanning rate. Pt rod as the counter electrode and RHE as the reference electrode were used in the same electrolyte chamber without membrane separation, Potential was corrected for IR compensation post measurement. No significant difference was observed for the 3 catalysts under RDE, due to the low limiting current caused by O_2 solubility in the electrolyte.

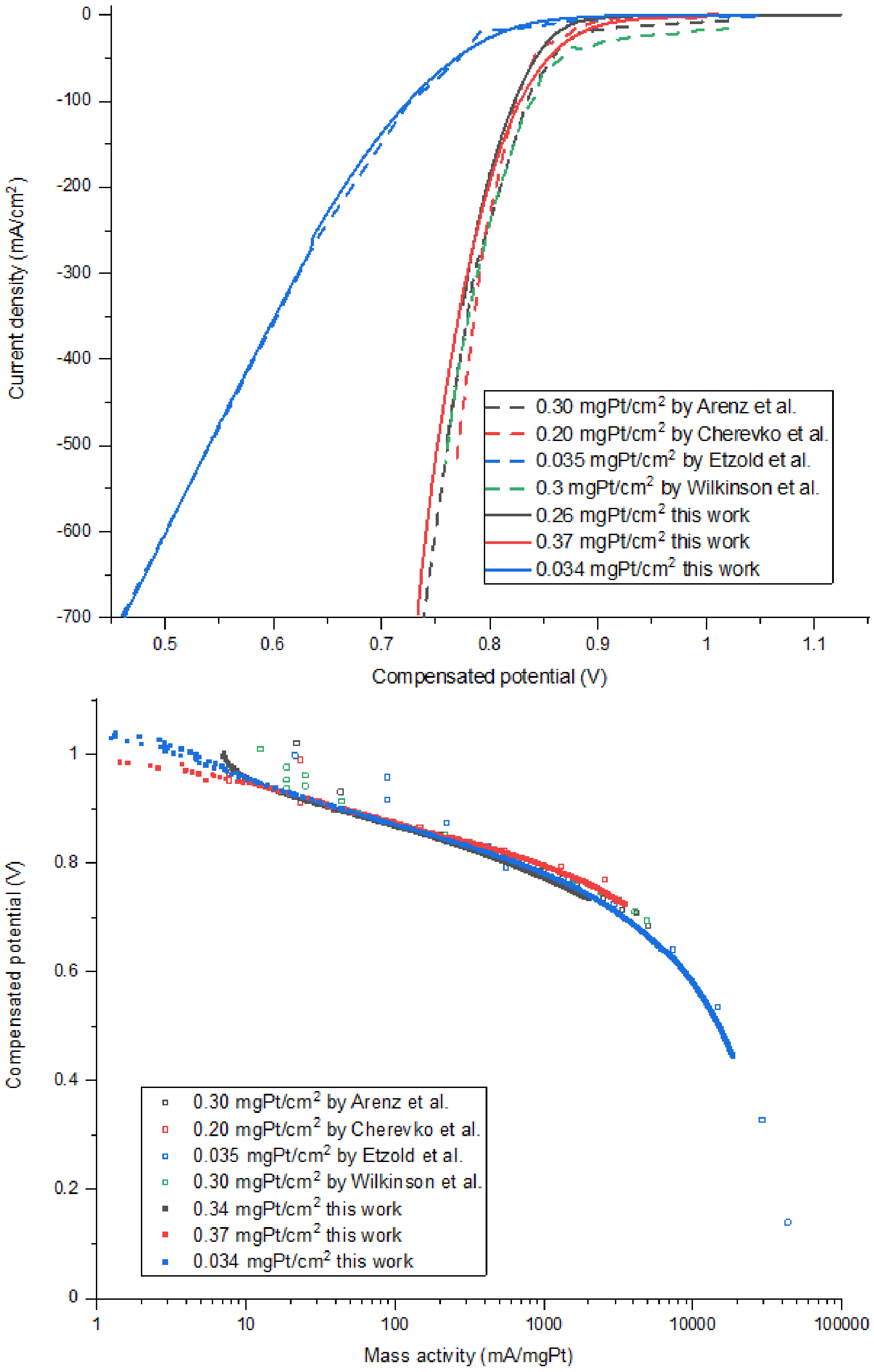

Figure S8. Benchmarking of the half -cell using HiSPEC4000 catalysts showing good agreement of the tested cell with other reported similar systems. Home measurements were done in 1M $HClO_4$ electrolyte at room temperature with 200 mL/min oxygen flow and 10 mV/s scanning rate. Literature results were extracted from respective publications.[123]

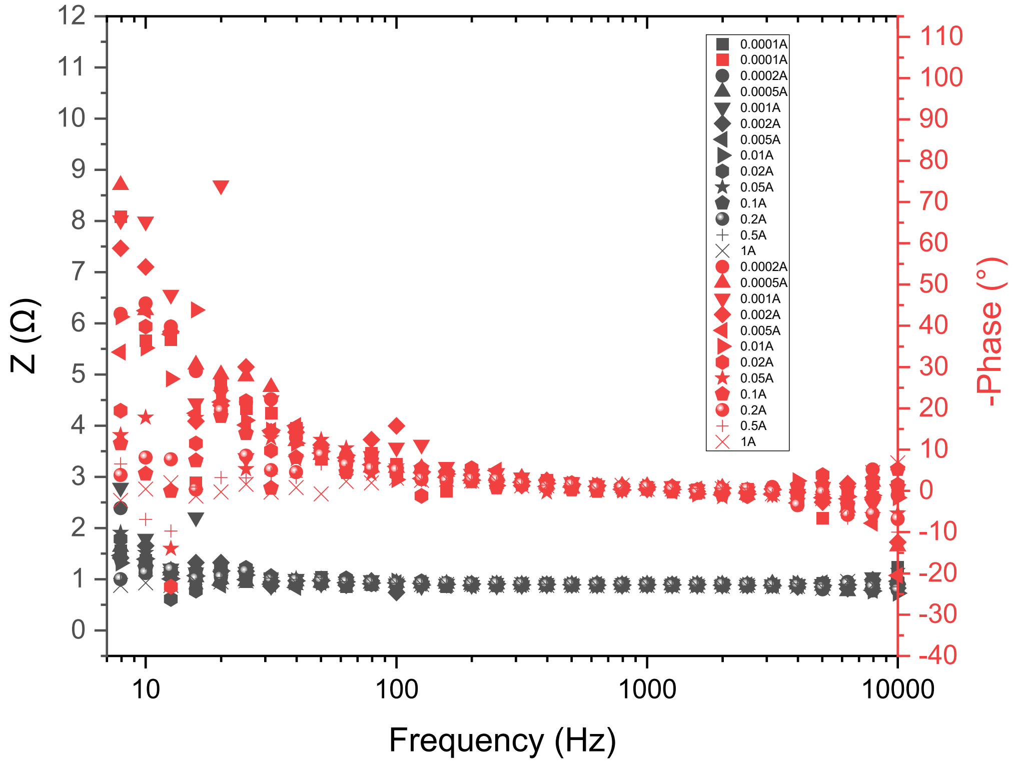

Figure S9. Bode plot of the impedance spectroscopy measurements from 1 mA to 1A indicating no R_u changes (catalysts area of 2.01 cm^2).

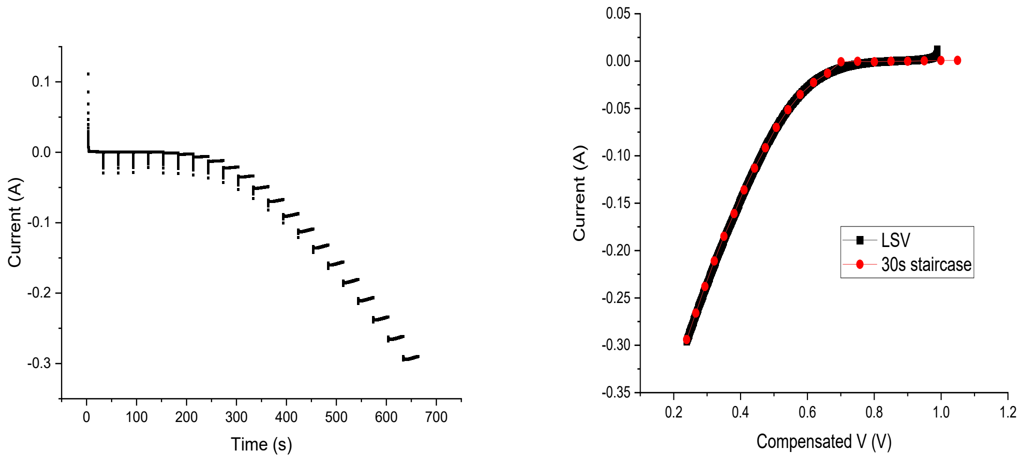

Figure S10. Comparison of chronoamperometry and linear sweep voltammetry measurements, showing no differences observed with the two different measurement protocols.

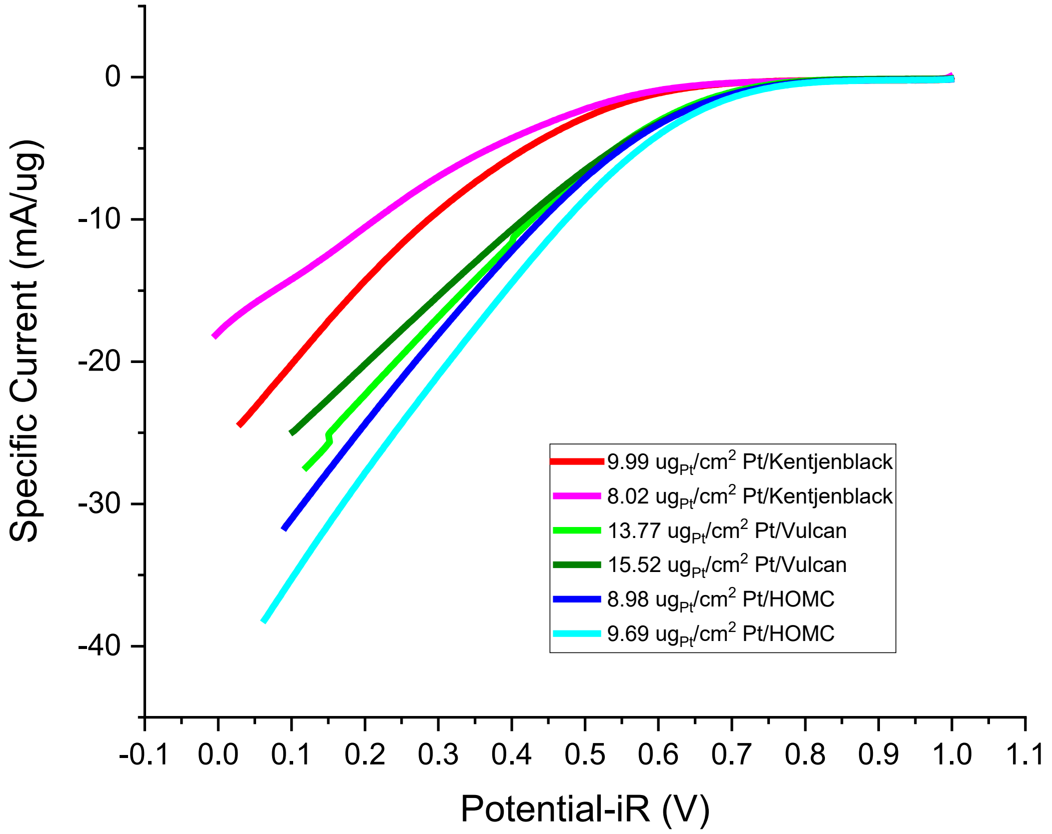

Figure S11. O_2 reduction reaction polarisation curve for the three different catalysts. Measurements were done in 1M $HClO_4$ electrolyte at room temperature with 200 mL/min oxygen flow and 10 mV/s scanning rate. Average of the two electrodes were used to generate Figure 3 in the main paper.

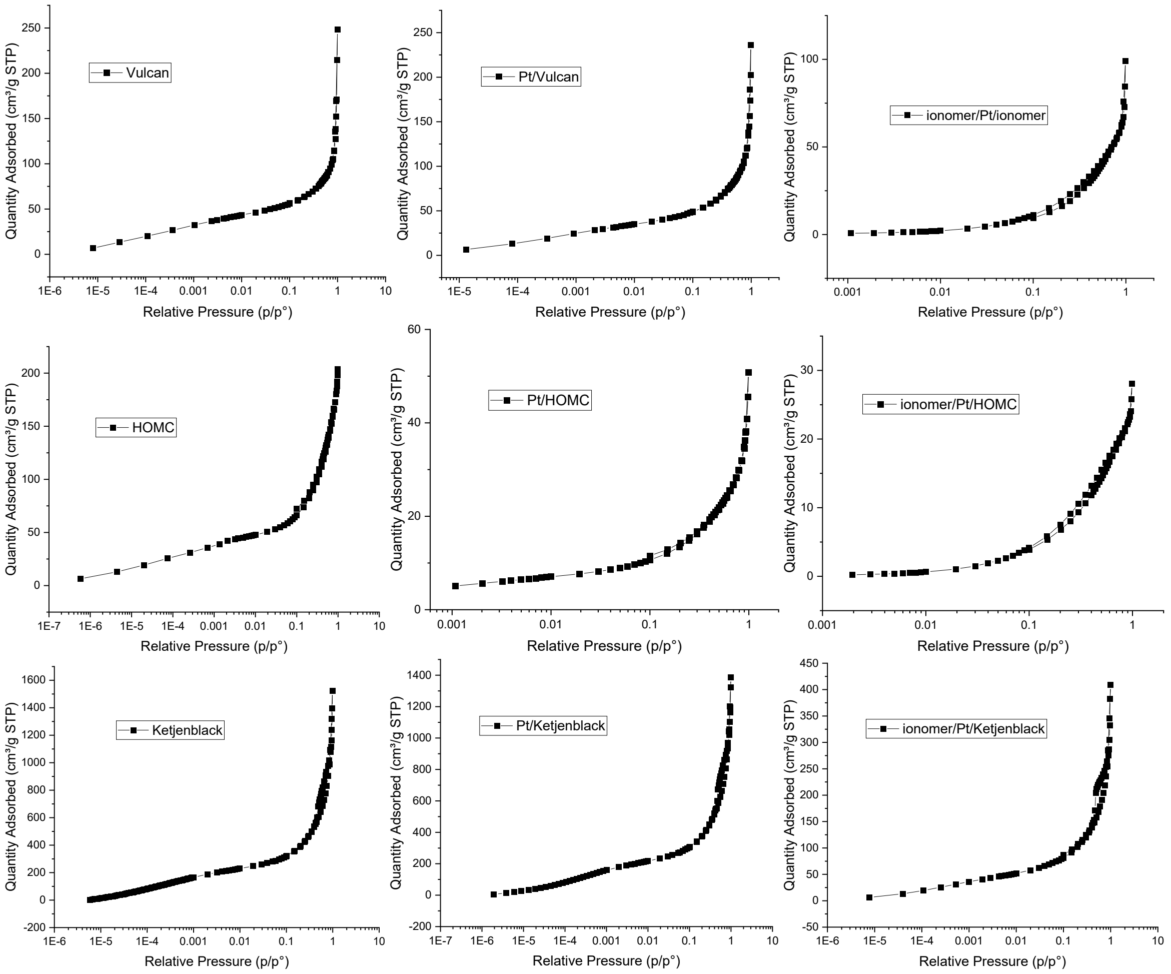

Figure S12. N_2 sorption data plotted with $\log(p/p^o)$ scale, supplementary to the isotherm data in Figure 2 and Figure 4.

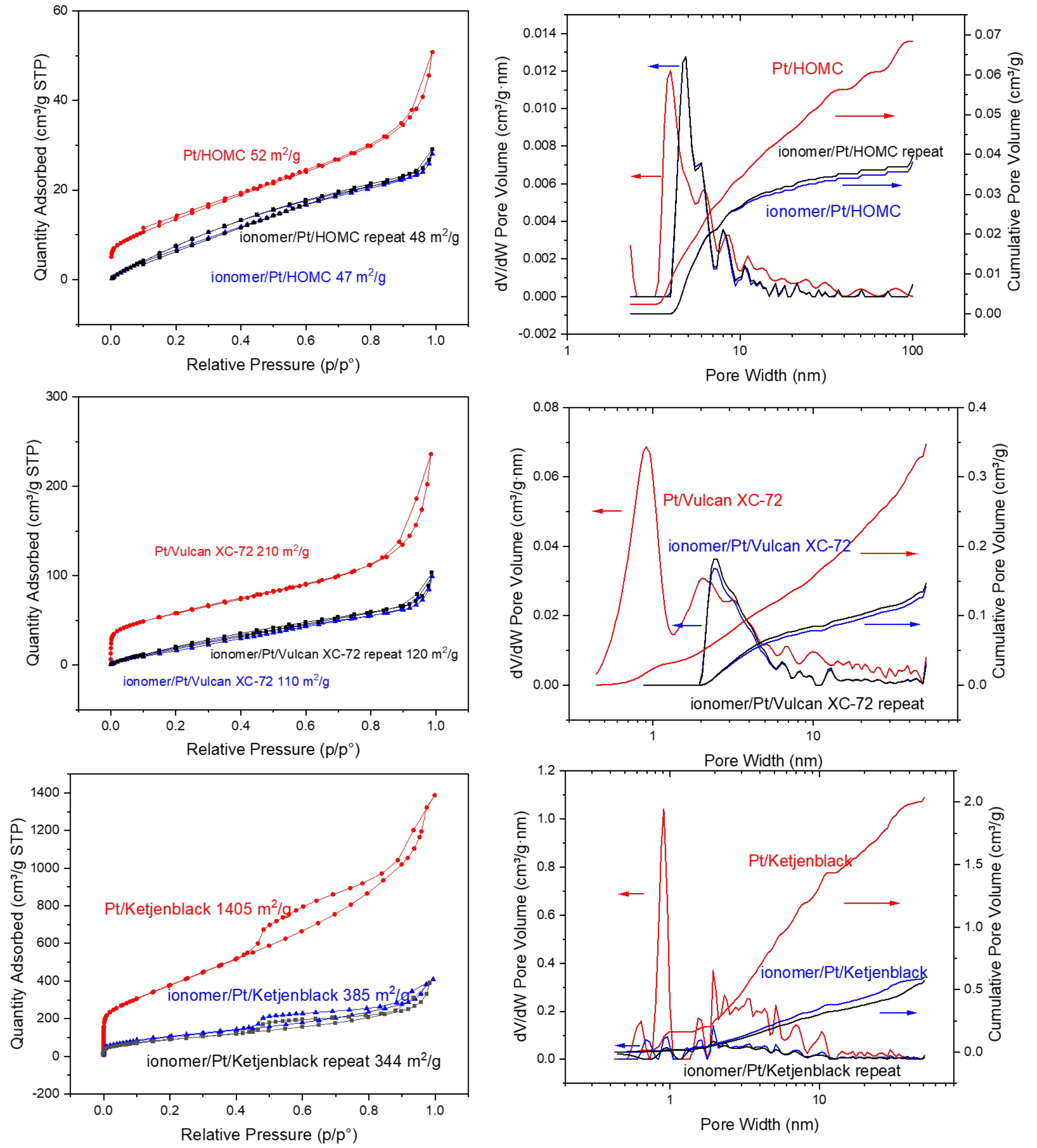

Figure S13. N_2 sorption of repeated tests for ionomer-containing samples, showing excellent reproducibility of the tests.

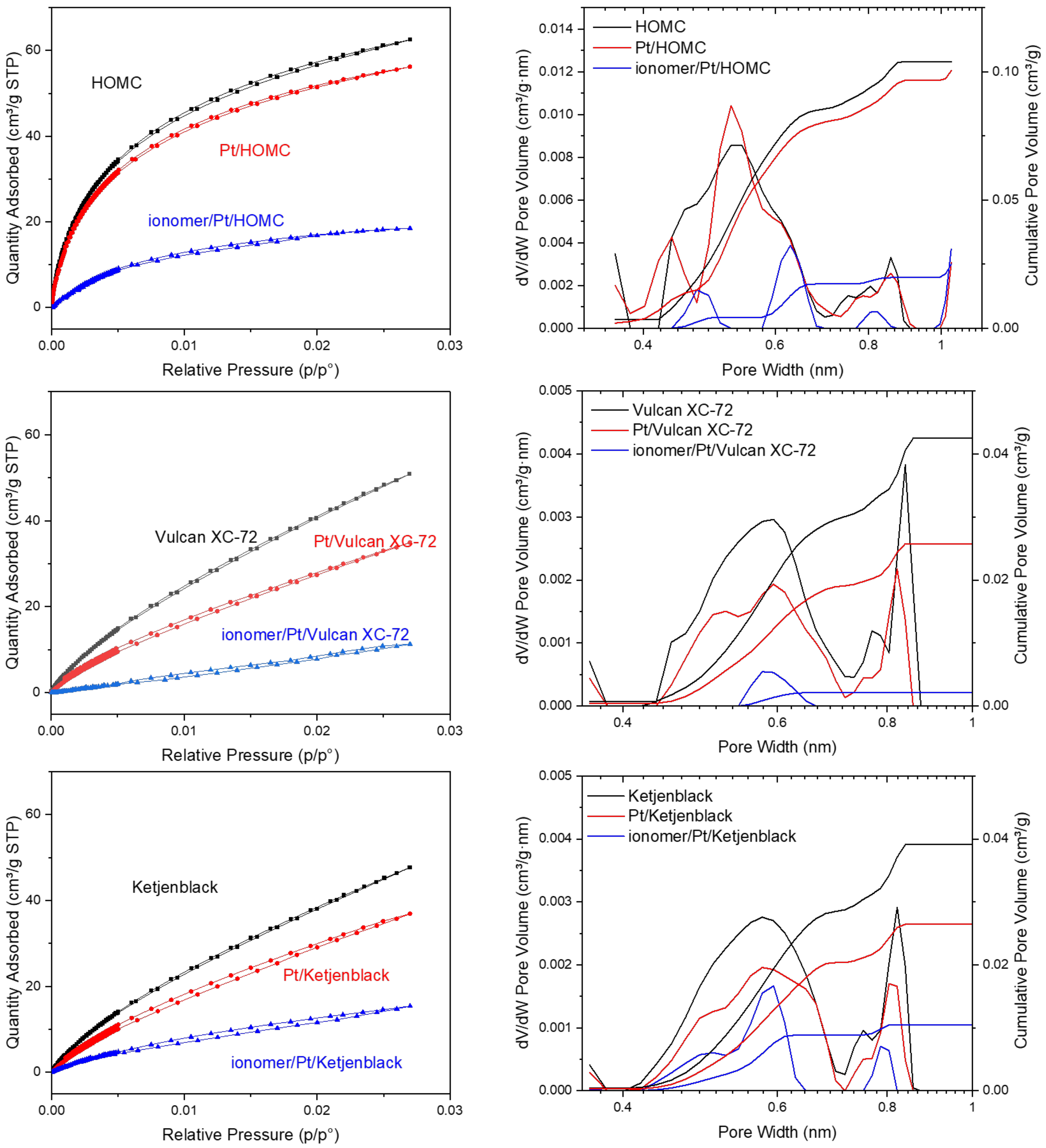

Figure S14. CO_2 sorption isotherms and pore width distributions, showing the difference of porosity below 1 nm caused by the introduction of Pt nanoparticles and the ionomer.

Table S2. White line intensity change with potential variations.

Potential vs RHE (V)	Pt L_3 edge white line intensity change[a]			Pt L_2 edge white line intensity change[a]		
	Pt/HOMC	Pt/Ketjenblack	Pt/Vulcan XC-72	Pt/HOMC	Pt/Ketjenblack	Pt/Vulcan XC-72
0.6	0.00244	0.00168	0.00640	-0.00128	-0.00490	0.00272
0.7	0.00641	0.00502	0.00869	0.00050	-0.00451	0.00056
0.8	0.00494	0.00527	0.01172	0.00098	-0.00324	-0.00234
0.9	0.01214	0.00825	0.01915	0.00562	-0.00133	0.00384
1.0	0.02238	0.02011	0.03615	0.01097	0.00622	0.01485

[a] Values were calculated by subtracting white line intensity at 0.5 V.

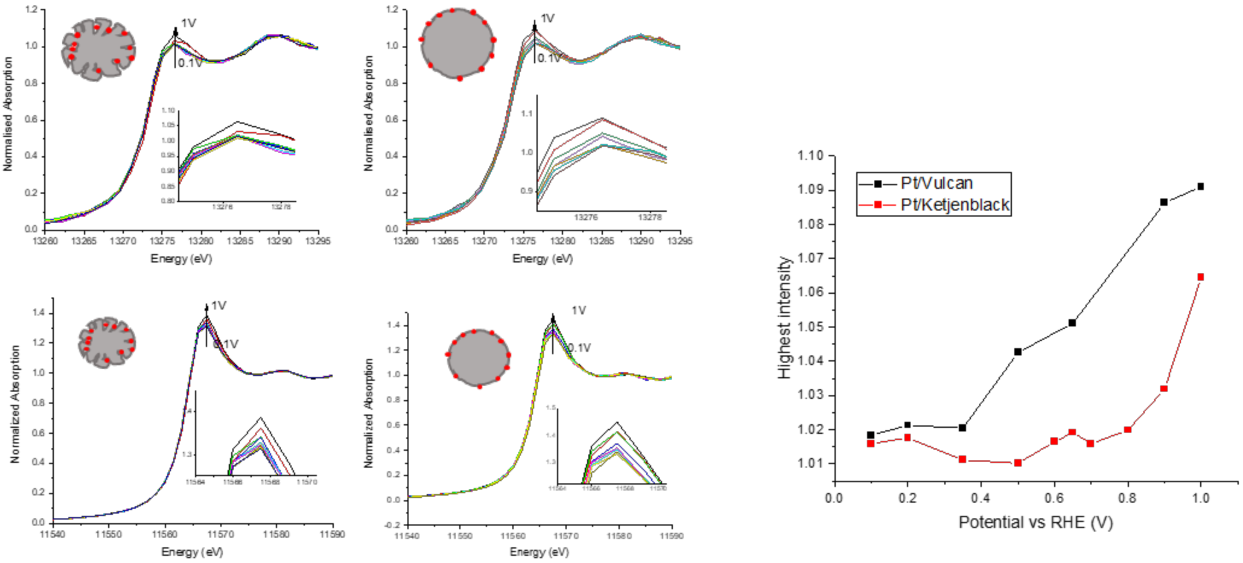

Figure S15. XANES results obtained from XMaS, ESRF showing the same observation for Pt/Vulcan and Pt/Knetjenbalck.

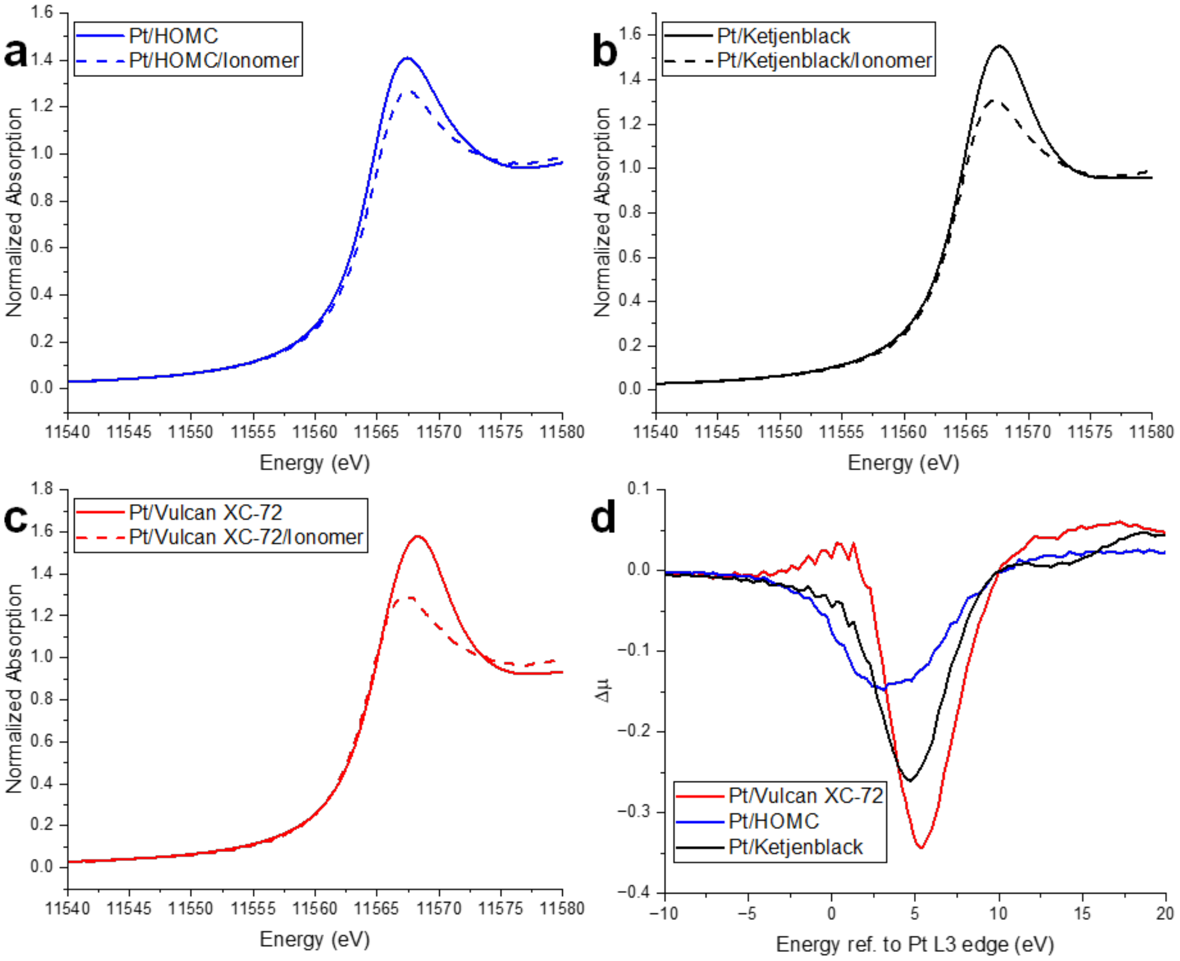

Figure S16. XANES spectra at Pt L_3 edge for catalysts with and without ionomer addition: (a) Pt/HOMC, (b) Pt/Ketjenblack and (c) Pt/Vulcan XC-72. (d) $\Delta\mu$ XANES obtained by subtracting normalized absorption for catalyst without ionomer from that for catalyst with ionomer.

As depicted in Figure S16d, the influence of ionomer on the catalysts varies, following the order of Pt/Vulcan XC-72 > Pt/Ketjenblack > Pt/HOMC. This trend aligns with our model, suggesting that Pt/Vulcan XC-72 has the most intimate contact with the ionomer, significantly affecting its electrochemical environment. These findings offer insightful observations into the distribution and impact of ionomer across different catalyst supports.

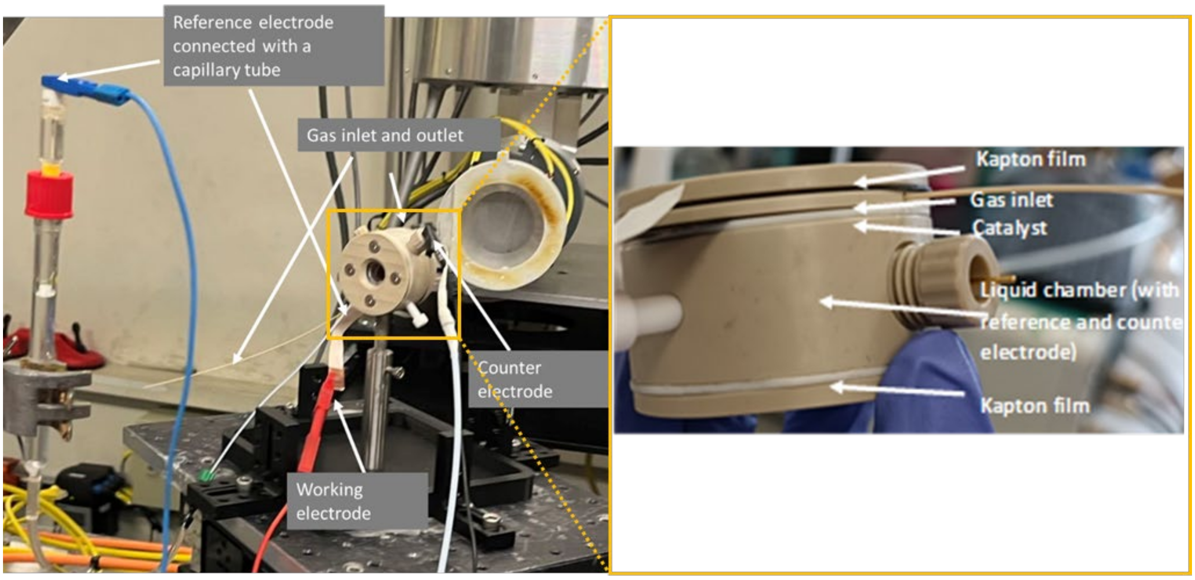

Figure S17. in-house designed gas-diffusion-electrode half-cell for operando XAS measurement.

Sample	Approx Beam Size (μm)	Approx Flux (ph/s)	Worst case time on Sample (HH:MM)
Pt/HOMC	100 x 100	4.3 x10^11	08:35
Pt/Vulcan	100 x 100	4.3 x10^11	07:53
Pt/ Ketjenblack	100 x 100	4.3 x10^11	09:55

Table S3. Summary of Beam Conditions During XANES Measurements

This table details the experimental beam conditions for XANES spectroscopy conducted on various catalyst samples. The approximate beam size indicates the focused beam dimensions at the sample position. The flux values, sourced from Diamond Light Source B18 Si (111) during commissioning, have been adjusted based on ion chamber (I0) absorption to provide an approximation of the flux at the sample location. The 'Worst case time on sample' represents the maximum exposure duration for each sample, calculated based on the longest interval between the start times of sequential scans.

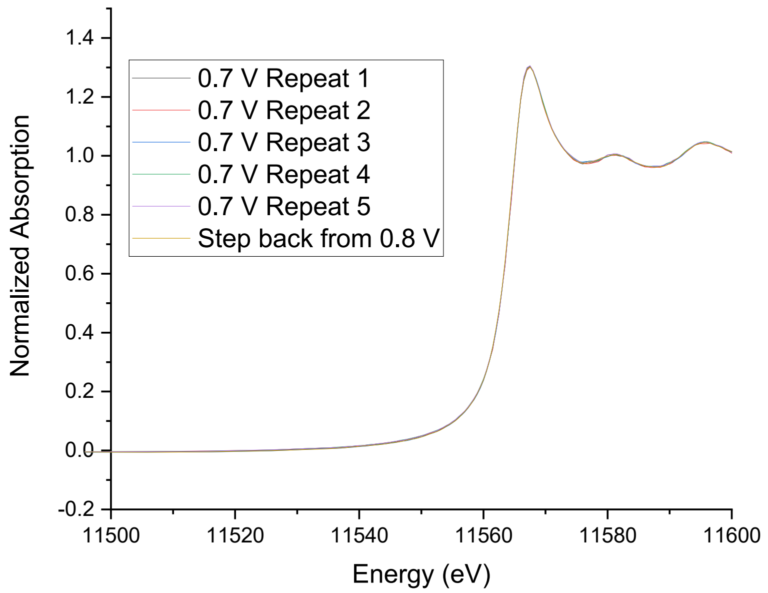

Figure S18. Pt L_3 edge absorption data collected for Pt/HOMC at 0.7 V, including 5 repeats collected after stepping up from 0.6 V and 1 repeat collected after stepping back from 0.8 V. The overlapping spectra show excellent reproducibility of the tests and confirmed the absence of beam damage to the sample.

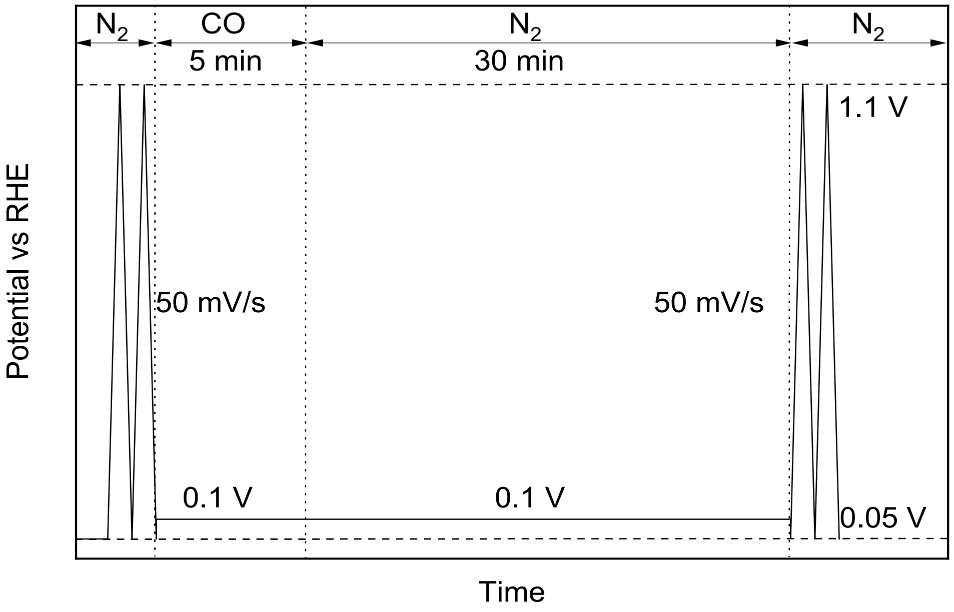

Figure S19. Potential profile and cathode gas compositions in the measurements of ECSA via CO stripping.

References

1 B. A. Pinaud, A. Bonakdarpour, L. Daniel, J. Sharman and D. P. Wilkinson, Key Considerations for High Current Fuel Cell Catalyst Testing in an Electrochemical Half-Cell, *J. Electrochem. Soc.*, 2017, **164**, F321–F327.

2 K. Ehelebe, D. Seeberger, M. T. Y. Paul, S. Thiele, K. J. J. Mayrhofer and S. Cherevko, Evaluating Electrocatalysts at Relevant Currents in a Half-Cell: The Impact of Pt Loading on Oxygen Reduction Reaction, *J. Electrochem. Soc.*, 2019, **166**, F1259–F1268.

3 N. Schmitt, M. Schmidt, G. Hübner and B. J. M. Etzold, Oxygen reduction reaction measurements on platinum electrocatalysts in gas diffusion electrode half-cells: Influence of electrode preparation, measurement protocols and common pitfalls, *J. Power Sources*, 2022, **539**, 231530.
